# Venom-gland transcriptomics and venom proteomics of the Tibellus oblongus spider

**DOI:** 10.1038/s41597-023-02703-0

**Published:** 2023-11-22

**Authors:** Yuliya Korolkova, Alexander Mikov, Anna Lobas, Elizaveta Solovyeva, Alexey Surin, Yaroslav Andreev, Mikhail Gorshkov, Sergey Kozlov

**Affiliations:** 1 Department of Molecular Neurobiology, Shemyakin–Ovchinnikov Institute of Bioorganic Chemistry RAS, 16/10 Miklukho-Maklay Str., 117997 Moscow, Russia; 2 Scientific Research Institute for Systems Biology and Medicine, Scientific Driveway, 18, 117246 Moscow, Russia; 3 V.L. Talrose Institute for Energy Problems of Chemical Physics, N.N. Semenov Federal Research Center for Chemical Physics, RAS, 38 Bld. 2, Leninsky Pr., 119334 Moscow, Russia; 4 Department of Molecular and Chemical Physics, Moscow Institute of Physics and Technology (National Research University), 9 Institutsky Per., 141700 Dolgoprudny, Russia; 5 Moscow Institute of Molecular Medicine, Sechenov First Moscow State Medical University, 8 Bld. 2, Trubetskaya Str., 119991 Moscow, Russia

**Keywords:** Proteome informatics, Peptides

## Abstract

The *Tibellus oblongus* spider is an active hunter that does not spin webs and remains highly underinvestigated in terms of the venom composition. Here, we describe venom glands transcriptome and venom proteome analysis for unveiling the polypeptide composition of *Tibellus oblongus* spider venom. The resulting EST database includes 1733 records, including 1263 nucleotide sequences with ORFs, of these 942 have been identified as toxin-coding. The database of peptide sequences was built based on of the transcriptomics results. It contains 217 new toxins, 212 of them were detected in the *T. oblongus* venom by the proteomics.

## Background & Summary

*Tibellus oblongus* (Walckenaer, 1802) belongs to the Philodromidae family and is distributed throughout the Holarctic ecozone. *T. oblongus*, as well as all other species of the Tibellus genus, is an active hunter that does not spin webs, but actively chases for preys.

For a long time, natural venoms have been valuable sources of biologically active compounds, and the venom of spiders is one of the most prospective in the rating list^1,2^.

The Bioproject NCBI server currently contains only transcriptomic data for Australian funnel-web spiders *Hadronyche infensa* (PRJEB6062). This analysis indicated that the venom contains more than 3,000 peptides and proteins divided into 32 superfamilies, 120 proteins are annotated. Nucleotide sequences coding toxins also are presented in two projects: the first project – PRJDB7399 “1,000 Spider Project provides de novo transcriptome assembly of spiders sampled from around the world” – contains transcriptome assembly complemented with long read sequencing for 56 species; the second one – SRX135055 “Araneae_mtgenomes_2011” contains mitochondrial genomes of different spider species. More than 4000 sequences are published as proteins on NCBI and a huge amount of non-processed transcripts are still hidden in transcriptomics data.

Technological progress has led to fast growth of OMICs approaches and their combinations. A combined proteomic, transcriptomic and genomic approach was used to study a number of peptides and proteins in different organisms including venomous animals. But the tendency to produce big data for further analysis can lead to incorrect results for proteins like peptide toxins that could differ in only a single point mutation. We have developed several methodologies for toxins retrieving from nucleotide sequence before^3,4^ and applied them to the sea anemone and spiders data processing^5,6^.

Early we described two insectotoxins from the venom of *T. oblongus* spider: peptide ω-Tbo-IT1^7^ was purified from the spider venom and peptide Tbo-IT2^8^ was predicted by cDNA analysis of venom glands transcriptome and confirmed by proteomic analyses using the LC-MS and MS/MS techniques. Here, we describe combined analyzes of transcriptome and proteome to clarify peptide composition of the *T. oblongus* venom.

## Methods

### Animal handling

The crude *T. oblongus* venom was purchased from Fauna Laboratories, Ltd. (Almaty, Republic of Kazakhstan). Only female spiders were collected in the nearby Almaty region. Venom and venom glands were obtained as described earlier^7,8^. Briefly, venom glands were extricated pair wise under Mustcam 1080 P Full HD USB-microscope, dissected from several specimens and frozen in liquid nitrogen until sample preparation. We activated toxins expression by a preliminary milking procedure (one week before RNA isolation). Crude venom for analysis was obtained from 13 female spiders by repetitive electrostimulation with intensive feeding between electrostimulations.

### EST library construction, sequencing, and data processing

Total RNA from venom glands was extracted as early described^8^. Total RNA was extracted from venom glands with SV Total RNA Isolation System (Promega, Madison, WI, USA). The yield and purity of RNA were determined at a Nanodrop ND-1000 spectrophotometer (Thermo Fisher Scientific, Waltham, MA USA), and RNA integrity was verified by the RNA Integrity Number (RIN) using Bioanalyzer 2100 (Agilent Technologies, Santa Clara, CA, USA). The PCR-based cDNA library was created using the SMART cDNA library construction kit (Clontech, Mountain View, CA, USA) and competent E. coli One ShotTOP10 cells (Thermo Fisher Scientific, Waltham, MA, USA). Plasmid DNA was sequenced using ABI Prism 3730xl automatic DNA sequencer (Sanger technique) with BigDye Terminator version 3.1 cycle sequencing kit (Thermo Fisher Scientific, Waltham, MA, USA). Basic operations with polynucleotide sequences such as Open reading frames (ORF) detection, translation of the ORFs to proteins were proceeded using in-house scripts. Signal peptide coordinates were found using SignalP algorithms. Pro-peptide length was detected using in-house scripts that takes into consideration so-called quadruplet motifs ‘XXKR’ and ‘XXRR’ located before the first residue of mature peptide.

Local protein BLAST (pBLAST) was used for homology identification, while CD-Search was utilized for conservative domains identification in proteins. Single-residue distribution analysis (SRDA) was used to elucidate mature polypeptide structures among sequences transformed initially using the key residue Cys and the termination translation symbol - SRDA (‘C.’). The 9 structural motifs covered all structural features of spider venom polypeptides were employed for the analysis (Table 1).

**Table 1:** The pattern of the search motifs.

Query	Cys distribution formula^§^	impact
Cys#1	C6C*CC*C*C#.	534
Cys#2	C6C*CC*C1C*C1C#.	5
Cys#3	CnC*CC*C*C#. (n≠6)	361
Cys#4	CnC*CC*C1C*C1C#. (n≠6)	0
extCys#2	C#C*CC*C1C*C1C	1
extCys#1	C#C*CC*C*C	25
partCys#1	C*CC*C*C#.	15
partCys#2	C*CC*C1C	6

The last column shows the impact of each motif on the number of overall sequences retrieved.

^§^special symbol used: #—any digit (0–9), *—gap in the search line,. —termination translation symbol encoded by the genes’ stop codon.

Both MS Excel and Origin 7.0 were exploited for general data analysis and plots generation. All in-house scripts were either built-in Excel Visual Basic macroses or short Python 3.0 scripts.

### Venom peptide fraction purification and peptide digestion

Venom Peptide Fraction was obtained by separation of 1.6 mg of the whole venom, dried, reduced with 5 mM dithiothreitol (DTT) for 30 min at 60 °C in 50 mM ammonium bicarbonate buffer and alkylated by 15 mM iodoacetamide (IAA) as early described^8^. The resultant protein content (25–30 µg per sample) was digested with trypsin (Promega, Madison, WI, USA), endoproteinase Lys-C (Promega, Madison, WI, USA), endoproteinase Asp-N (Roche, Mannheim, Germany), and endoproteinase Glu-C (Promega, Madison, WI, USA). The enzymes were added at the ratio of 1:75 w/w to the total protein content and the mixture was incubated overnight at 37 °C for trypsin, Lys-C and Asp-N and at room temperature for Glu-C, digests were performed according to the protocols^9^. After the reaction was stopped with 3% formic acid (final concentration), the samples were dried up using CentriVap micro IR Vacuum Concentrator (Labconco Corporation, Kansas City, USA) at 45 °C. Dried peptides were stored at −45 °C until the LC-MS/MS analysis.

### LC-MS/MS analysis of peptide digests

The chromatographic separation of the samples was performed using Easy-nLC system (Thermo Fisher Scientific, Waltham, MA, USA) and two home-made columns (150 mm × 75 µm) with stationary phases Aeris™ 1.7 µm PEPTIDE XB-C18 100 Å (Phenomenex, Torrance, CA, USA) and Aeris™ 3.6 µm WIDEPORE XB-C18 300 Å (Phenomenex, Torrance, CA, USA). The second phase has wider pores and therefore is more appropriate for separation of bigger molecules such as peptides arising from digestion with Asp-N, Glu-C and Lys-C. Analytical separation was performed using mobile phase A - 0.05% formic acid, 0.05% trifluoroacetic acid (TFA) in water, and mobile phase B - 0.05% formic acid, 0.05% TFA, 10% water in acetonitrile (ACN), with gradient elution at a flow rate of 0.3 µL/min. Two gradients with different lengths were applied for peptide separation. For the first column with smaller pores, the concentration of mobile phase B had an increase from 0% to 60% over the first 60 min, then to 90% B over 10 min followed by washing with 90% B for 20 min. For the second column longer and flatter gradient was applied, the concentration of mobile phase B was increasing up to 50% in 100 min, then up to 90% in 20 min followed by washing for 15 min.

The mass spectrometric analysis of all samples was performed on a high-resolution Orbitrap Elite mass spectrometer (Thermo Fisher Scientific, Waltham, MA, USA) in data-dependent acquisition mode with two sets of parameters used with shorter and longer gradients respectively. For both analyses, MS scan resolution was set at 60 K with maximum injection time of 100 ms, 2 m/z isolation window width was used to isolate precursor ions, and fragmentation spectra were acquired at resolving power 30 K. The other parameters were lined up with the length of LC gradient and presumable higher mass of peptides (Table 2).

**Table 2:** Mass spectrometer settings for peptide measurement corresponding to two sets of LC conditions.

	100 Å pore column/Short gradient	300 Å pore column/Long gradient
Automatic gain control (MS)	5 × 10^5^	1 × 10^6^
Dynamic exclusion time, s	10	30
Top N (number of sequential MS/MS scans)	10	7
Maximum injection time (MS/MS), ms	200	250
Automatic gain control (MS/MS)	5 × 10^4^	2 × 10^5^
Normalized collision energy	28	30

### Quality control and data analysis

The quality control of proteomics data was performed using viQC program^10^. The protein sequence database in FASTA format was built based on transcriptomics results, containing 217 toxins, for 88 of which more than one isoform was given. The mass spectrometry raw data were converted to mzML format using Msconvert software^11^. The identifications were obtained using Identity search engine (https://github.com/levitsky/identipy)^12^. The digest specificity was set corresponding to the proteases used (some having low digestion efficiency) with no more than 4 missed cleavages limitation. Carbamido methylation was set as a fixed modification of cysteine residues and as a variable modification of methionine, histidine, lysine, and tryptophan residues due to the high concentration of alkylation agent used. The C-terminal residue amidation was also set as a variable modification. The precursor and fragment mass tolerances were set at 10 ppm and 0.02 Da, correspondingly. The post-search filtering to 1% of false discovery rate (FDR) at peptide-spectrum match level as well as data analysis, comparison, and visualization was performed using in-house scripts based on Pyteomics library^13^. The spectra considered dubious or suspicious based on the automated analysis were checked manually.

## Data Records

The generated database includes 1733 ESTs which were submitted to figshare repository (10.6084/m9.figshare.21842034.v4)^14^. The repository includes four Excel Dataset files named Tibellus_ESTdata.xls (contains EST data processing), Tibellus_mature_toxins.xls (contains predicted mature toxins), Tibellus_venom.xls (contains proteomics results), Tibellus_toxins_GenBank.xls (list of toxin precursor proteins submitted to GenBank).

**Fig. 1:**
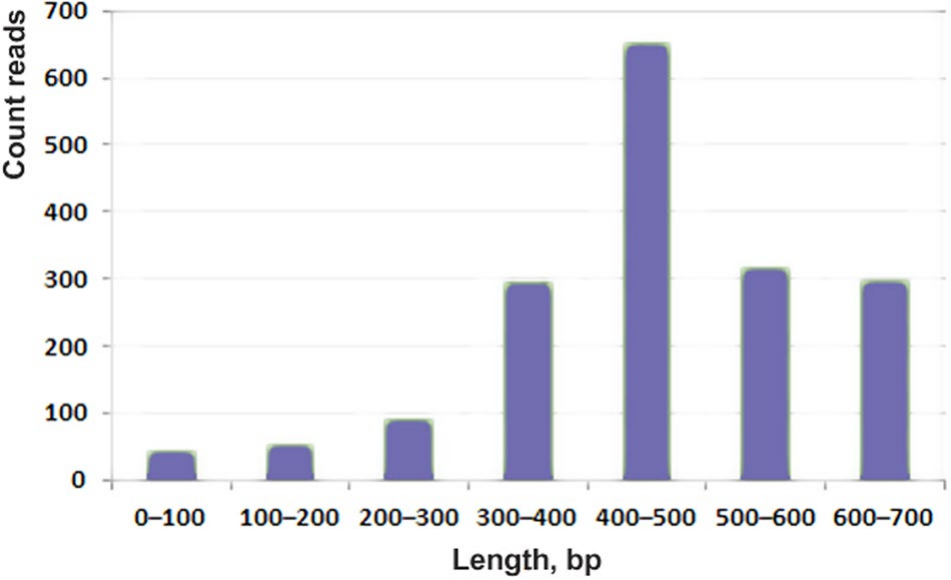
Distribution of single reads by length in the dbEST *of T. oblongus*. The prevalent sequence group had a 400–500 bp length, which is enough to encode a precursor protein larger than 100 amino acids (a.a). Thus, the quality of data obtained was sufficient for protein search in raw sequences without preliminary contigs formation, which is a common practice for NGS data processing. In this research, each read was considered as an independent transcript and translated sequences were analyzed.

The database contains cDNA sequences obtained from the glands of several spider species collected at the same place and time. Double-stranded cDNA was preliminary fractionated in agarose gel and only fraction >700 bp was cloned and sequenced. This size is sufficient to determine by Sanger sequencing the full-size precursor of most spider toxins, which are proteins of interest in this project. The median reading length was 457 nucleotide residues (Fig. 1).

Data after verification were processed by scripts^15^ and ORFs were detected for 1263 nucleotide sequences. In total, 723 translated sequences having a stop codon at the 3′ terminus were identified as EST coded for full-length polypeptides. The final results are included in the file Tibellus_ESTdata.xls^14^. After removal of clones encoding identical CDS, 345 sequences encoding full-length spider toxin precursor proteins were submitted to GenBank (BankIt2675870: OQ473925 - OQ474269). The full list of accession numbers correlated with toxin’s names is in the file Tibellus_toxins_GenBank.xls^14^.

**Fig. 2:**
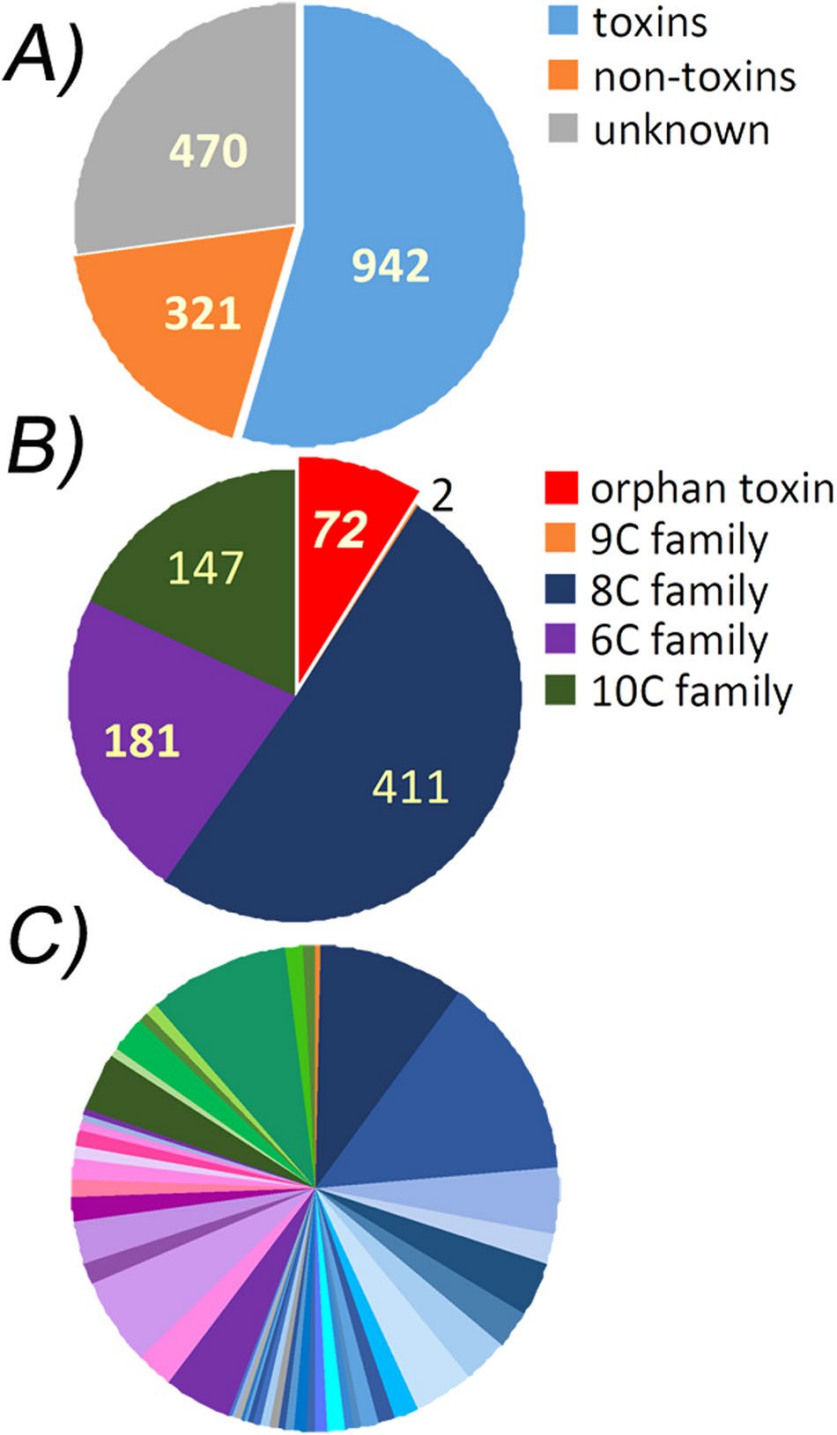
Distribution of venom glands EST of the spider *T. oblongus* by groups. (**A**) Number of toxins and other sequences in the database, (**B**) The distribution of orphan toxins and main toxins families, (**C**) The size of toxin subfamily, are colored in blue gamma for the main 8 C family, violet gamma for the main 6 C family, green gamma for the main 10 C family, and orange for the 9 C family.

Spider toxins in glands are expressed as a set of homologous sequences (families) that contain synonymous and non-synonymous substitutions along the mature chain. Individual sequence analysis (contigs free) allowed us to identify single mutations throughout precursor proteins sequences to count toxins variants in the venom, to group homologous peptides in family and subfamily, and to segregate housekeeping proteins (Fig. 2).

Among unrelated to toxins sequences, 316 were determined as valuable since BLASTx analysis revealed their similarity with fragments of known proteins with enzymatic, regulatory, structural, and other functions that were out of the study goal.

The amount of derived toxins’ sequences was 942 out of 1733 as shown on Fig. 2A. Further, only the toxins group was analyzed. All toxins were divided among four main families, depending on the number of cysteine residues in the sequence and primary structure homology (Fig. 2B).

The protein sequence set in FASTA format was built for mature toxins’ sequences, considering possible C-terminal modifications that occur in spider toxins (available in the file Tibellus_mature_toxins.xls^14^). This set of proteins sequences was used for components verification in a natural venom sample by proteomics.

The mass spectrometry proteomics data have been deposited to the ProteomeXchange Consortium via the PRIDE^16^ partner repository with the dataset identifier PXD040219^17^. Proteomics data include raw LC-MS/MS runs, as well as the search results and the database used in fasta format. To obtain raw mass spectrometry data, the venom peptide fraction was digested with enzymes with different specificities: Lys-C, Glu-C, and Asp-N. The high similarity of toxins’ sequences within families manifested in the replacement of one residue made it difficult to define the composition of toxins in the sample using a single enzyme for fragmentation.

**Fig. 3:**
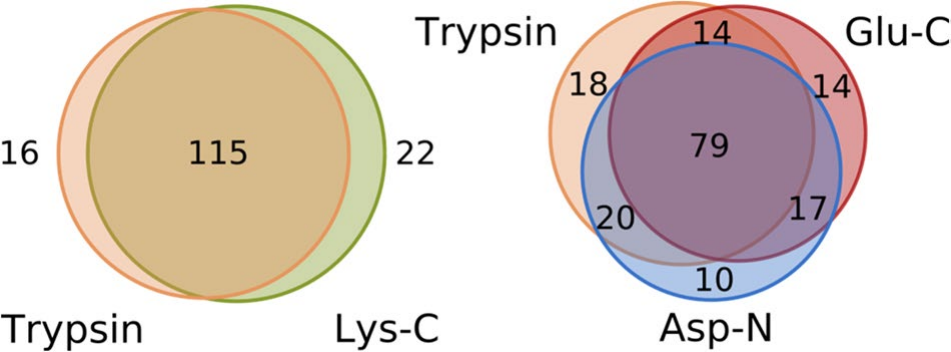
Comparison between the sets of toxins uniquely identified in proteomics analyses of four digests obtained using enzymes with different specificity. The Venn diagrams show the intersections between the sets of polypeptide toxins identified by at least one unique peptide in the samples digested by proteases with different specificities: (left panel) trypsin and Lys-C, (right panel) trypsin, Glu-C, and Asp-N.

A polypeptide toxin from the venom was considered unambiguously identified if at least one of its unique proteolytic peptides was identified. The comparison of identifications obtained using different proteases was performed only for unambiguously identified peptides and is presented in Fig. 3 in the form of Venn diagrams. The identifications from trypsin and Lys-C digests are close to one another that can be explained by similar specificity of these enzymes, while two other proteases add approximately equal number to the pool of uniquely identified toxins. Out of 217 polypeptides in the database, trypsin digest resulted in 131 identifications, while the combination of results gave 178 toxins identified at proteome level by at least one unique peptide (82% of sequences). Thus, the use of various proteases allowed us to uniquely identify 36% more toxins compared to the classical hydrolyzation by trypsin. We did not find any unique peptides for 39 toxins from the database, but this fact does not exclude their presence in the sample. In total, peptide evidence was found for 212 of 217 toxins, but 4 of them had insufficient level of sequence coverage. The Tibellus_venom.xls^14^ file includes data of venom’s peptides endorsement.

As a result, we provided structural information about the 217 novel polypeptides and detected more than 200 compounds in the venom of *T. oblongus* spider.

## Technical Validation

### Sequences processing pipeline

**Fig. 4:**
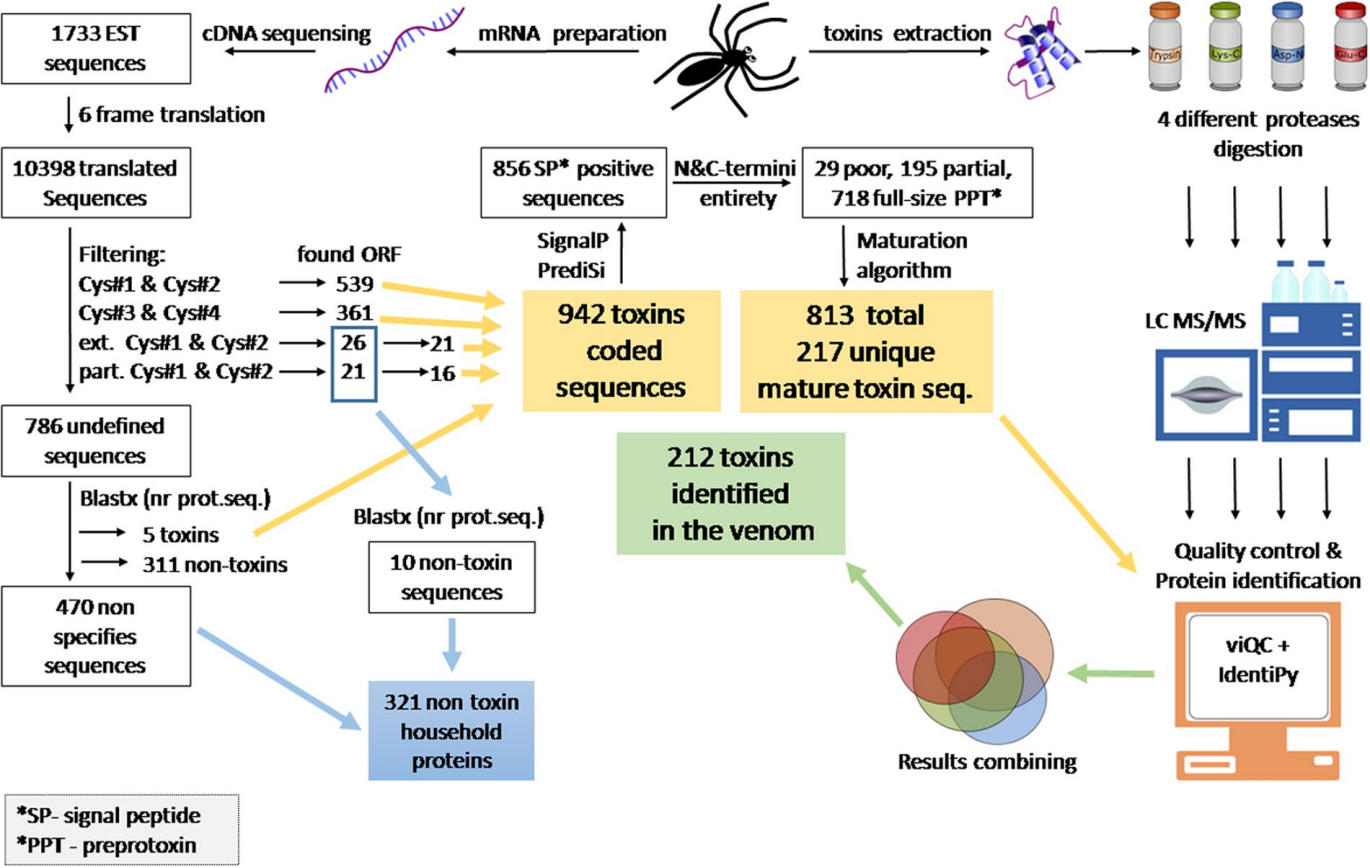
Pipeline of *T. oblongus* venom compounds elucidation.

The overall analytic scheme of toxins determination in the venom of *T. oblongus* spider as a combination of proteomics and transcriptomics method is presented on Fig. 4. The translation of each individual non-combined single read in 6 reading frames led to the preliminary database formation consisting of 10,398 amino acid sequences. ORFs were determined by in-house scripts for protein sequence analysis and the remaining 5 reading frames were automatically removed from the data set, since the second protein coding into alternative reading frames is unknown in the case of spiders.

A step-by-step process was used to maximize the effectiveness of toxins structures detection. At first, spider toxins were determined by presence into translated sequences of the general cysteine residues distribution patterns also known as structural motifs^6,15^. Queries based on motifs Cys#1 and Cys#2 gave almost identical results due to a formula overlapping, and Cys#3 and Cys#4 gave the same results. In addition to general spider toxins motifs, we used extended and partial motifs, which consider structures with incomplete sequence at C- or N-terminus. However, almost all toxins encoded in the database were successfully determined by general motifs (Cys#1- Cys#4 in Table 1).

The search based on the presence of structural motif resulted in 947 initial reads encoding toxins or toxin-like polypeptides (Table 1). The remaining part of 786 initial nucleotide sequences was analyzed by the BLASTx algorithm against non-redundant database, that led to the identification of additional 316 EST shared homology to known proteins. ESTs encoding for toxins could be identified as toxins using BLAST, but we did not analyze their homology with known structures, except for sequences obtained using the extCys#2, extCys#1, partCys#1 and partCys#2 motifs. These reads were verified by the BLASTx algorithm, and 10 sequences were removed from the group of toxins. After BLAST analysis, 470 original nucleotide sequences remained unidentified (these were mostly sequences with multiple errors or of insufficient length). Thus, the main part (942 sequences) was assigned to the group of toxins, and 321 sequences were assigned to the group of non-toxins (Fig. 2A).

Toxins are secreted molecules that have a characteristic signal peptide signature in the primary structure of precursor. Most spider toxins also contain propeptide after signal peptide, which is cleaved posttranslationally^18^,^19^. The toxin group was analyzed for the presence of signal peptide signature by two specialized services: http://www.predisi.de/predisi/). In total, a signal peptidase processing site was found for 856 sequences analyzed. The nearest methionine about 20–27 residues upstream from this site was considered as a start codon of translation. The presence of stop codon was analyzed downstream to the processing site of signal peptidase. If both start and stop codons were detected successfully, the sequence was considered complete (unless no internal errors were found). Only 195 sequences were partial, and the most of the sequences coded for full-length precursors of toxins.

Mature sequences of toxins were determined by the previously described algorithm^3^, capable of detecting the specific motif for a precursor processing protease^20^. Additionally, we considered the possible activity of carboxypeptidases and peptidylglycine-alpha-amidating monooxygenase that could eliminate several amino acid residues at C-terminus of the peptide. Cleavage by C-terminal carboxypeptidases is difficult to predict unambiguously, so we included numerous variants of possible mature toxins in the final list for proteomics. The good quality EST coding for toxins (813 including repeated mature sequences) yielded 217 unique sequences of peptides. We used the proteomics approach to confirm the presence of these toxins in the sample of *T. oblongus* venom.

### Proteomics data collection and quality control

The transcriptomic analysis of the venom gland gave an understanding of its potential molecular constitution, however, proteomics analysis was required to find out toxins that are actually translated as polypeptides. According to the transcriptome-based data on polypeptide sequences of *T. oblongus* venom, there is a high level of homology (191 of 563 theoretical trypsin peptides with no missed cleavages and length more or equal to five amino acid residues are non-unique, i.e. correspond to more than one toxin). To overcome this ambiguity, LC-MS/MS analysis was performed not only for trypsin digest, but also for three digests prepared using enzymes with different specificity: Lys-C, Glu-C, and Asp-N. Data are available via ProteomeXchange (http://www.proteomexchange.org/) with identifier PXD040219^17^. The use of proteases with different specificity allowed to obtain more unique peptides, therefore getting unambiguous identification of the toxins. The longer peptides produced by enzymes targeting rare residues in general have the higher probability of being unique. The overall workflow of proteomic analysis of the venom is shown in Fig. 4.

**Fig. 5:**
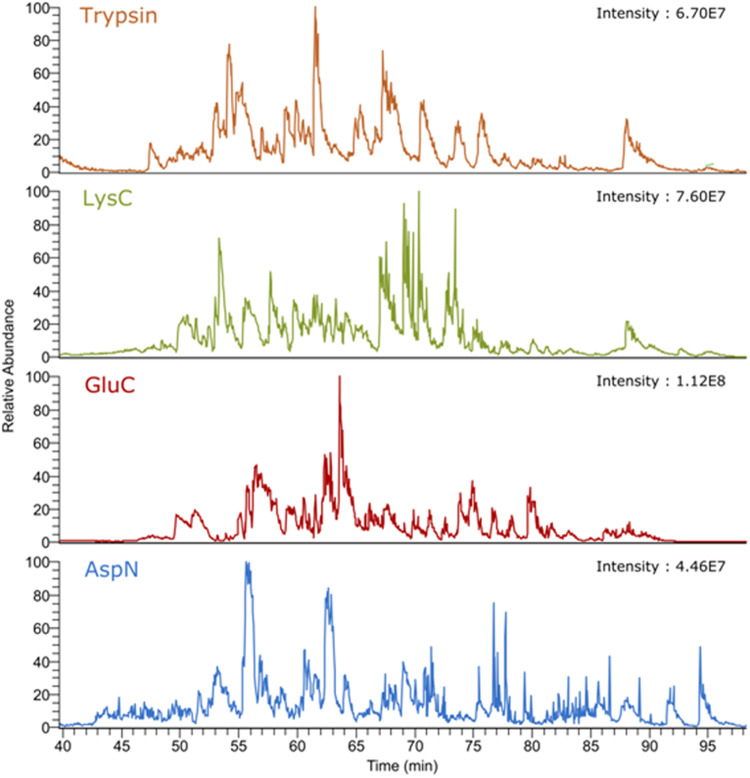
Base peak chromatograms of four digests of *T. oblongus* venom using enzymes with different specificity and standard conditions for LC-MS analysis.

Figure 5 shows base peak chromatograms for four venom digests measured under standard LC-MS conditions with comparable maximum intensities (4.5 × 10^7^ to 10^8^) and different elution profiles throughout the gradient time range, illustrating the digestion efficiency and different specificity of the four proteases.

**Fig. 6:**
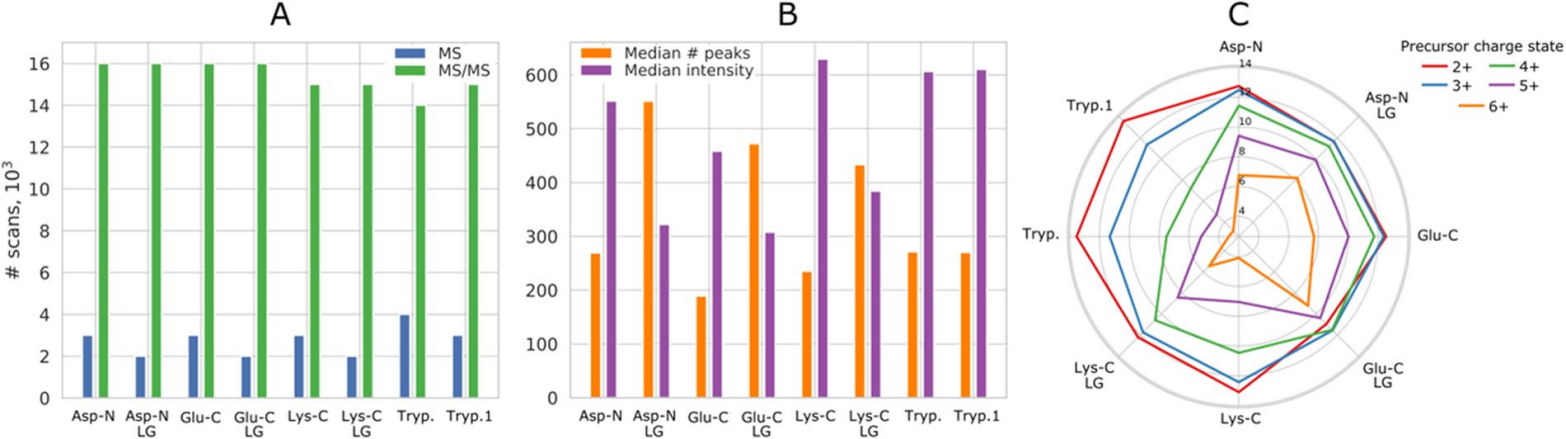
The quality control of eight LC-MS/MS analyses of the spider venom digests obtained using proteases with different specificity (Asp-N, Glu-C, Lys-C and trypsin). For all proteases except trypsin analyses were done with standard LC-MS/MS settings and adjusted ones (denoted as LG for long gradient), for trypsin digest two replicas were measured under the same conditions. (**A**) Number of MS and MS/MS scans measured in each analysis. (**B**) Median number of peaks in MS/MS scans and their median intensities. (**C**) Number of fragmentation spectra of precursor ions with a particular charge state in log2 scale.

The quality control was performed by viQC program with input files in mzML format. For all eight analyses, the following parameters were controlled: the number of MS and MS/MS scans, their injection time and acquisition time, the distribution of precursor intensities and charge states, as well as the number of sequential fragmentation spectra and their quality. The identification process is based on fragmentation spectra, and therefore their number is the first critical parameter in proteomic experiments. In our data, the mean number of MS/MS spectra is roughly 15.5 thousand with a standard deviation of 500 spectra (Fig. 6A). Interestingly, the lowest number was obtained for a classical tryptic digest, which may reflect a difference in the initial protein abundance. The further step in data quality control is the MS/MS spectra quality itself, which was evaluated by calculation of the median number of peaks and their median intensities (Fig. 6B)^21^. It should be noted that the noise peaks filtration is unnecessary in this approach. Both the median number of peaks and their median intensities are similar for all digests measured under standard conditions. However, the number of peaks in spectra was significantly higher in the analyses with the parameters adjusted for bigger peptides acquisition, which may reflect higher collision energy as well as higher masses of measured molecules. The number of precursor ions with charge state exceeding 3+ is expectedly lower for tryptic digests (Fig. 6c). Moreover, the number of fragmentation spectra measured for precursor ions with high charge states (5+ and 6+) is notably higher for analyses in adjusted conditions, testifying to the proper experimental settings choice for the bigger peptides.

### Toxins identification

**Fig. 7:**
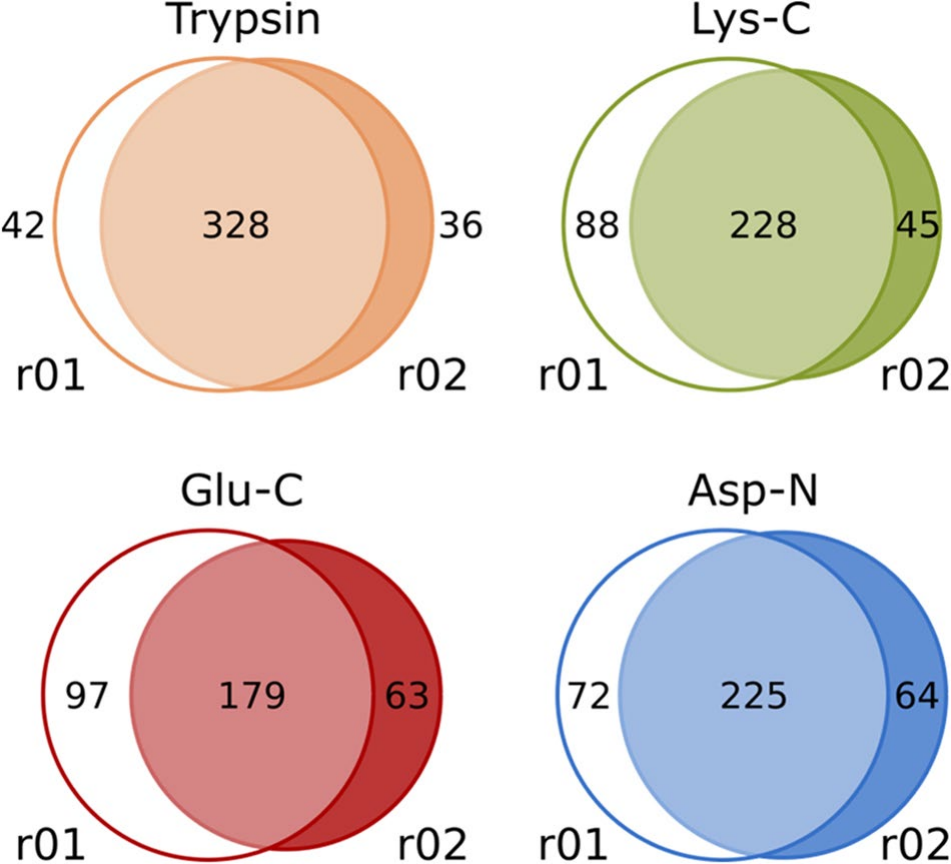
Venn diagrams of proteolytic peptides identified in two replicates of LC-MS/MS analysis of four venom digests. For trypsin, both replicates were analyzed under the same conditions; for other proteases, the second replicate was measured under conditions optimized for longer peptides.

The LC-MS/MS data was analyzed using IdentiPy search engine and filtered to 1% (FDR at peptide-spectrum match level). For Lys-C, Glu-C and Asp-N digests of *T. oblongus* venom second replicates of LC-MS/MS analysis were measured under conditions optimized for longer peptides, while the trypsin digest was analyzed in duplicate under standard conditions. For this reason, the identified proteolytic peptides for trypsin digest analysis replicates overlap much more than those for other proteases (Fig. 7). The gain in peptides total number clearly highlights the utility of using different chromatographic and mass spectrometric settings. However, for all proteases the number of peptides identified in first replicates is higher, and therefore both types of conditions were used in combination for the deep analysis of the sample.

**Fig. 8:**
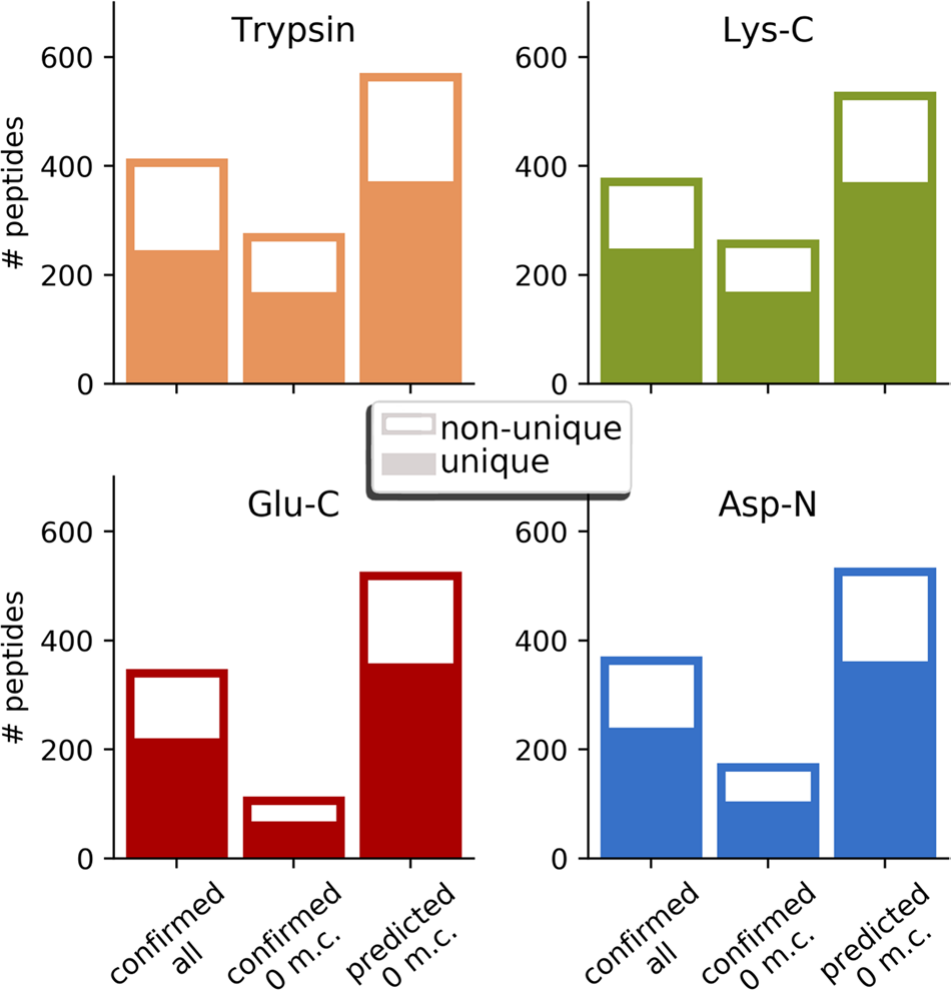
The numbers of unique and non-unique proteolytic peptides identified in the analyses of four digests of *T. oblongus* venom. To assess the confirmation rate of theoretical proteolytic peptides, only ones with length more than four residues and without missed cleavages (designated as 0 m.c. on the plot) were considered.

For further identification of toxins, both runs for each protease were united, and peptide coverage was determined. Because of the high homology between the toxins’ sequences mentioned before, a significant fraction of the identified proteolytic peptides were non-unique, i.e. shared between two or more sequences of toxins (Fig. 8). For trypsin digest, as expected, the fraction of non-unique peptides was higher than for the other enzymes (165 out of 410 peptides, or 40.2%), the lowest one for Lys-C (127 out of 375, or 33.9%) and similar for Asp-N and Glu-C, showing that corresponding peptides were in general longer than tryptic ones and therefore more likely to be unique. To assess the confirmation rate of the proteomics experiments, the numbers of unique and non-unique theoretical peptides with length more than four amino acid residues were calculated. Only peptides without missed cleavages were considered in this case not to overinflate the theoretical peptide set, and the confirmation rate was the highest for Lys-C protease (45% for unique and 55% for non-unique peptides) and the lowest for Glu-C protease (19% and 24%, respectively), partially because of the lower digestion efficiency of the latter protease resulting in higher rates of missed cleavages.

**Fig. 9:**
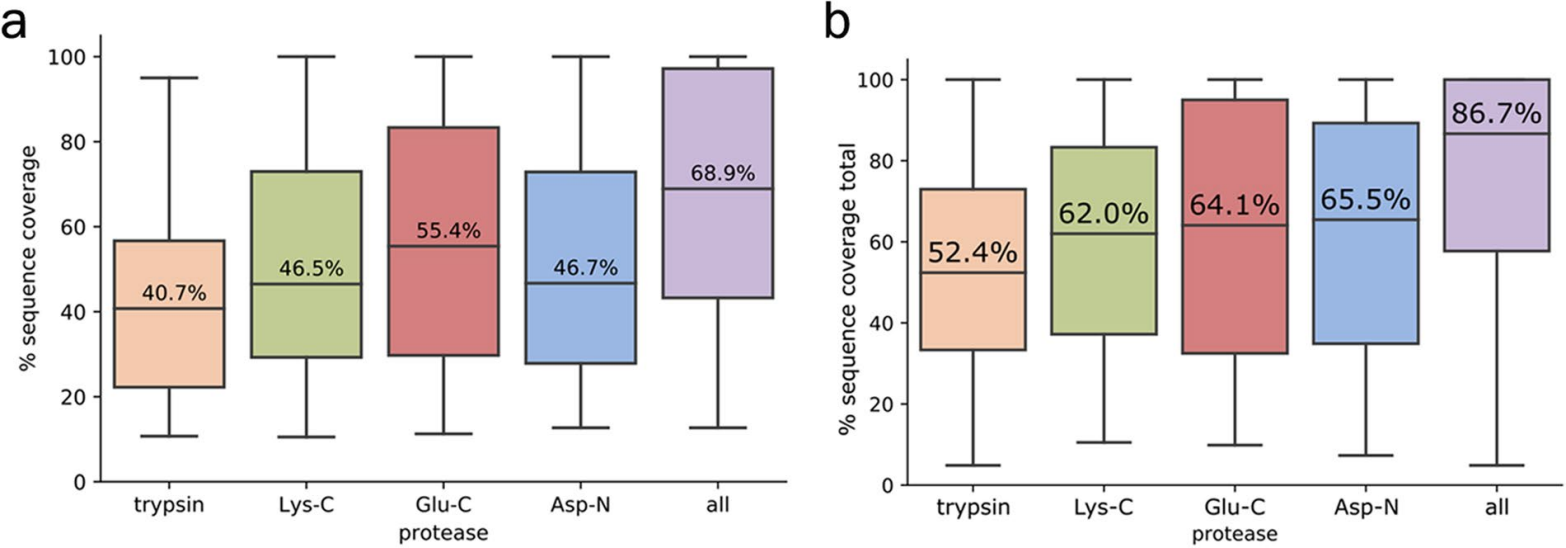
Box plots of sequence coverage of *T. oblongus* toxins (**a**) by unique and (**b**) by all peptides identified in the analyses of four digests by proteases with different specificity. The numbers in the boxes correspond to the median value of the sequence coverage of identified toxins.

For 88 out of 217 toxins in the database, several isoforms (different variants of peptide processing) were assumed from the transcriptomic analysis. For all of those, only one isoform was confirmed by unique peptides. If the identification was ambiguous, the isoform with higher sequence coverage was chosen. The last quantitative metric for estimation of the proteomics analysis depth was sequence coverage of the identified toxins by unique peptides. It was calculated as the ratio between the part of the sequence corresponding to the identified unique peptides and the length of the whole sequence. Figure 9a shows the box plots of sequence coverage by unique peptides identified in each of the digests by four proteases and in the combination from all of them. As trypsin cleavage is specific to two frequent amino acid residues (arginine and lysine), the peptides in the corresponding digest are shorter and less unique, therefore giving the lowest sequence coverage compared to the other enzymes. Figure 9b illustrates sequence coverage by all peptides, including both unique and non-unique ones.

We can conclude that, for spider toxins with a specific primary structure organization, the multienzyme approach applied herein resulted in a higher level of sensitivities to both quantity (more toxins identified) and quality (higher sequence coverage of the toxins). This implies a higher level of confidence in the venom analysis.

### Post translational modification

C-terminus amidation is a very common PTM for spider toxins that should be determined by the corresponding C-terminal peptide identification. Since amidation can be confused with isotope selection error, dubious peptide-spectrum matches were inspected manually. Peptide-spectrum matches of the amidated and not amidated variants of each unique and non-unique C-terminal peptide were counted, and all identified toxins grouped according to the amidation evidence are listed in the file Tibellus_venom.xls^14^. Based on the transcriptome analysis, amidation was predicted for 43 toxins. Proteomic analysis confirmed the modification of 15 toxins based on unique peptides, 13 ones were confirmed based on non-unique C-terminal peptide for five protein groups. Seven more predicted amidation sites were identified as ambiguous (there are mass spectra matching both modified and unmodified peptides), and for three toxins no amidation was shown in the investigated venom. The remaining five toxins sharing C-terminal amidation motif were not covered by proteolytic peptides, so PTM status of these toxins is undefined. Notably, no unambiguous amidation was found on any toxin besides the predicted sites.

### Multifamily organization of T. oblongus toxins

Considering proteomics data, it can be assumed that almost all toxins derived by transcriptomics actually presented in the venom of spider. Thus, the spider *T. oblongus* produces a lot of structurally distinct molecules (217 were identified in EST, 212 of them were found in venom). We divided them into 3 large families (6 C, 8 C and 10 C) and a small 9 C family by the number of cysteine residues in the peptide sequence. Additionally, toxins without homologues were found. Within families, we grouped highly homologous toxins into subfamilies.

**Fig. 10:**
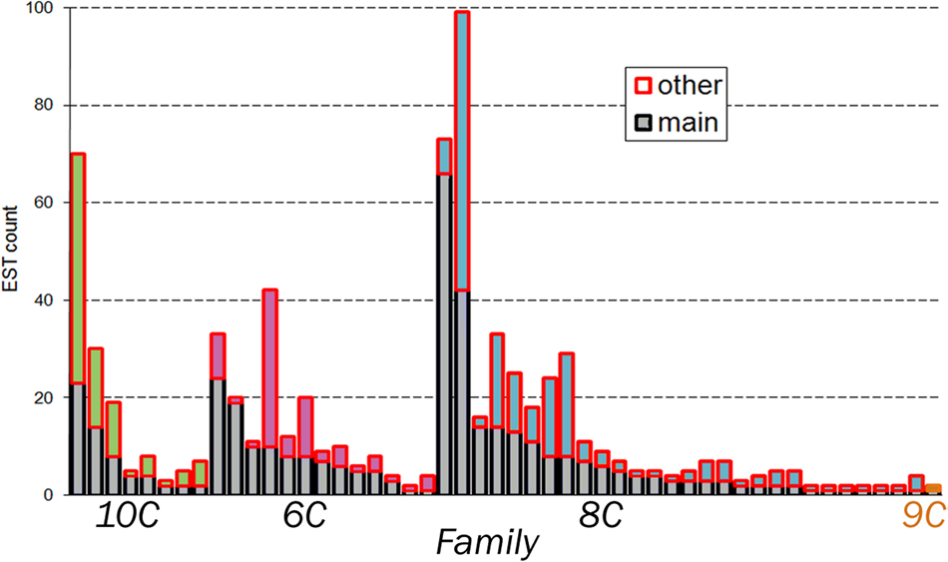
Number of sequence transcripts for the dominant sequence and homologue transcripts in different *T*. *oblongus* toxin subfamilies. Within the toxin subfamily, the dominant sequence is shown in gray. The content of homologue transcripts in individual subfamilies of the 10 C family is shown in green, the 6 C family in purple, the 8 C family in blue, and the 9 C family in yellow.

Highly homologous groups of toxins were previously reported for other spiders^22,23^ and it may be associated with the evolution of venoms. Typically, but not necessarily, there is a dominant sequence in a subfamily that outperforms other variants in the transcripts count (Fig. 10).

The main structural fold of cysteine-rich peptides of spider venom is the inhibitor cystine knot (ICK). The ICK fold was initially characterized by a three-stranded antiparallel beta sheet stabilized by three disulfide bonds C1–C4, C2–C5, C3–C6 where the bond C3-C6 passes through the characteristic ring formed by two other disulfide bonds and a peptide backbone^24^. Spider toxins with “canonical” ICK fold usually contain six cysteine residues, but a variant with 8 cysteine residues is also common.

**Fig. 11:**
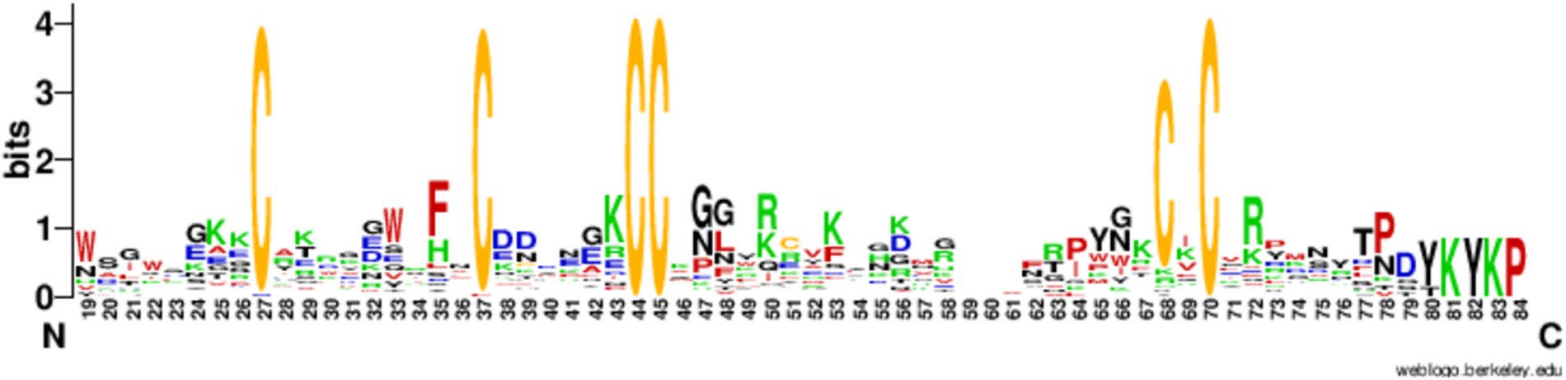
Consensus sequence of 45 toxins belonging to the family 6 C. The multiple alignment of all members was done in MEGA^25^ and presented using WebLogo - a web service for visualization of alignment results^26^.

The 6 C family of *T*. *oblongus* includes 45 toxins, divided by homology into 13 subfamilies (Fig. 11). We found a stable distribution of cysteine residues in the N-terminal and central parts of toxins’ sequences.

**Fig. 12:**
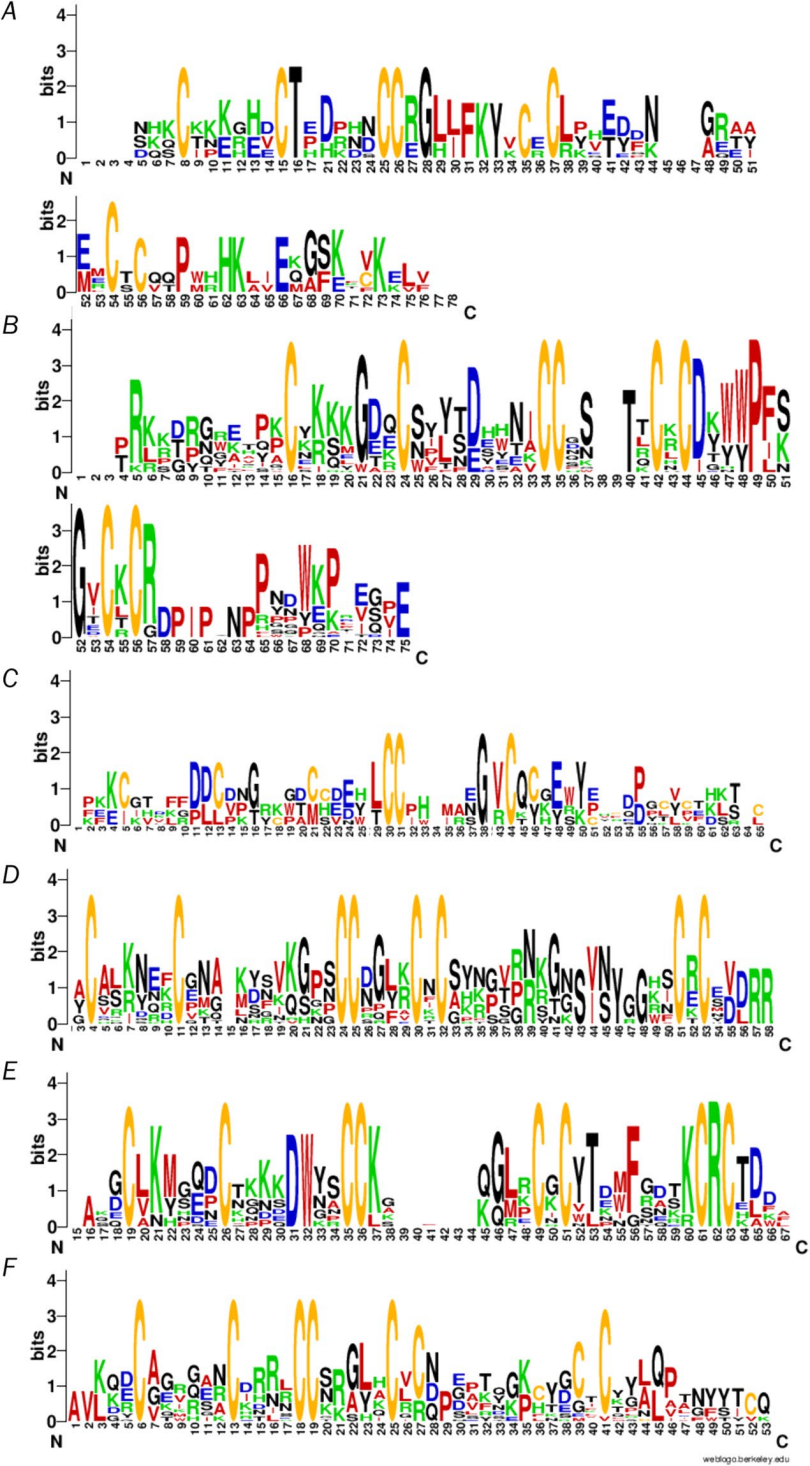
Consensus sequence of the family 8 C. The multiple alignment of 8 members (**A**), of 25 members (**B**), of 8 members (**C**), of 21 members (**D**), of 17 members (**E**), of 18 members (**F**) was done in MEGA^25^ and presented using WebLogo - a web service for visualization of alignment results^26^.

Members of the 8 C family, containing 8 cysteine residues, are the most common toxins in the *T*. *oblongus* venom. (see Figs. 2, 10). The 97 toxins of this family were divided into 28 subfamilies because they have highly variable lengths of peptide chains (minimum 36 a.a., maximum 71 a.a.) (Fig. 12).

The 9 C family contains only two toxins differed in first residue, which have an unbound cysteine residue.

**Fig. 13:**
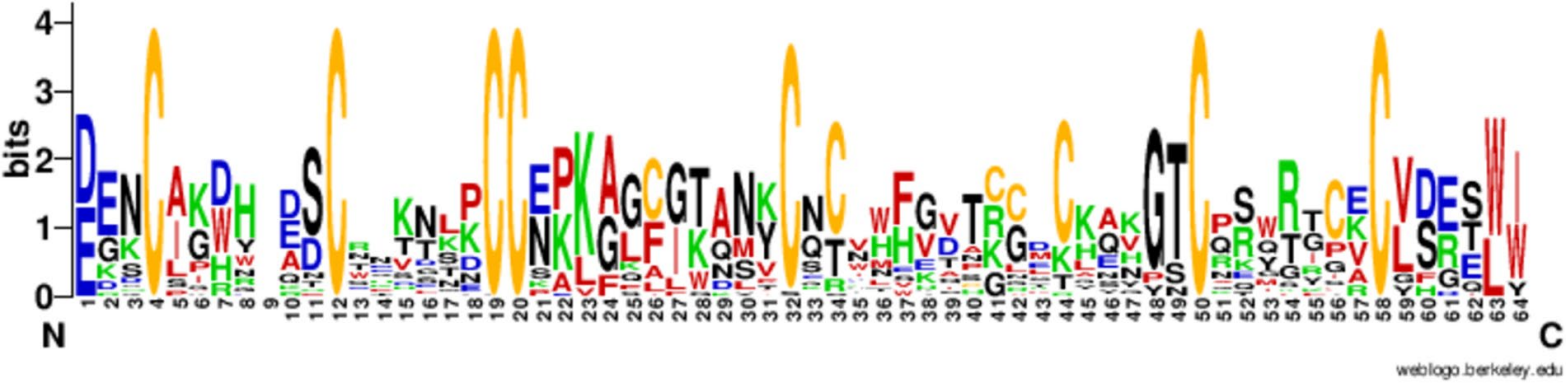
Consensus sequence of 35 toxins belonging to the family 10 C. The multiple alignment of all members was done in MEGA^25^ and presented using WebLogo - a web service for visualization of alignment results^26^.

A distinctive feature of the *T. oblongus* venom is the wide representation of unusual ICK peptides containing 10 cysteine residues. The 10 C family includes 35 different toxins, divided into 8 subfamilies with high homology within the subfamily (Fig. 13**)**. According to the alignment data, 10 C toxins have a stable configuration of seven cysteine residues in the sequence, all other positions in the sequence, with rare exceptions, are highly variable.

## Data Availability

The script code for toxins cDNA analysis can be accessed as supplementary materials to the article “The mining of toxin-like polypeptides from EST database by single residue distribution analysis”^15^. No special constants were used. Identity search engine^12^ is available at https://github.com/levitsky/identipy.
